# HDAC10 Is Positively Associated With PD-L1 Expression and Poor Prognosis in Patients With NSCLC

**DOI:** 10.3389/fonc.2020.00485

**Published:** 2020-04-21

**Authors:** Xiaomei Liu, Yuxi Wang, Rong Zhang, Ting Jin, Liangliang Qu, Qianwen Jin, Jiasu Zheng, Jiaqi Sun, Ziqing Wu, Linxi Wang, Tianxu Liu, Yinxu Zhang, Xiao Meng, Ying Wang, Ning Wei

**Affiliations:** ^1^Department of Oncology, The First Affiliated Hospital of Jinzhou Medical University, Jinzhou, China; ^2^Department of Medicine, Jinzhou Medical University, Jinzhou, China; ^3^Pharmacy Department, Dalian Hospital of Traditional Chinese Medicine, Daliang, China; ^4^Department of Surgery, The First Affiliated Hospital of Jinzhou Medical University, Jinzhou, China; ^5^Division of Hematology-Oncology, Department of Medicine, University of Pittsburgh School of Medicine, Pittsburgh, PA, United States; ^6^Cancer Therapeutics Program, UPMC Hillman Cancer Center, University of Pittsburgh, Pittsburgh, PA, United States

**Keywords:** NSCLC, HDAC10, PD-L1, pulmonary squamous carcinoma, adenocarcinoma

## Abstract

Currently, non-small cell lung carcinoma (NSCLC) is a major worldwide health problem. Meanwhile accumulating evidence indicates that histone deacetylase (HDAC) activation could induce PD-L1 expression in various types of cancer, especially in myeloma and B-cell lymphomas. Therefore, we hypothesized that high-level expression of HDAC10 is associated with PD-L1 induction and poor prognosis in patients with NSCLC. In total 180 NSCLC patients receiving complete pulmonary resection and systematic lymph node dissection were enrolled from April 2004 to August 2009. The patients with integrated clinicopathological records were followed up. The expression level of HDAC10 and PD-L1 in tissue samples was determined by immunohistochemistry. We observed that HDAC10 expression in lung cancer tissue is significantly higher than that in corresponding para-cancer tissue. Moreover, HDAC10 expression positively correlated with the expression level of PD-L1 (*r* = 0.213, *P* < 0.05) in NSCLC patients. Subgroup, multivariate analysis showed that the expression level of HDAC10 can be an independent prognostic factor and high-level expression of HDAC10 indicated poor overall survival for pulmonary carcinoma (*r* = 0.540, *P* < 0.001). Our findings suggest that the expression level of HDAC10 is positively associated with PD-L1 expression and may predict the outcome of patients with NSCLC.

## Introduction

Due to advances in operation techniques and novel treatments (targeted therapy and PD-L1 immunotherapy) for patients with non-small cell lung carcinoma (NSCLC), the prognosis of NSCLC is improved. However, the 5-years survival rate of NSCLC patients is still <20% and NSCLC remains a major health problem worldwide. Therefore, it is urgent to discover novel targets to regulates the carcinogenesis and metastasis of NSCLC and to develop new therapeutic agents for the treatment of NSCLC (1, 2). Recently, accumulating evidence indicates that abnormal regulation of acetylation processes plays a vital role in lung carcinogenesis. For example, histone deacetylases (HDACs) and histone acetyltransferases (HATs) can significantly change the nucleosome conformation of tumor cells through post-translational modifications of the N-terminal tails of core histones (3, 4). However, the expression level and clinical characteristics of HDAC10 in NSCLC tissue is not clear. Tumor-associated PD-L1 (B7 homolog 1, B7-H1) can block tumor-specific T cell-mediated immunity through inducing apoptosis of T cells, suppressing the secretion of cytokines and disturbing the function of activated T cells (5, 6). Moreover, recent findings suggested that activation of HDACs could induce PD-L1 expression in various types of cancer, especially in myeloma and B-cell lymphomas (7–9). Thus, we hypothesized that high-level expression of HDAC10 is closely associated with PD-L1 expression and poor prognosis of patients with NSCLC.

Herein, we evaluated HDAC10 and PD-L1 expression in patients with NSCLC. Furthermore, the correlation of HDAC10 and PD-L1 was analyzed. HDAC10 expression and poor prognosis in patients with NSCLC receiving pulmonary resection was also investigated.

## Materials and Methods

### Patients

From April 2004 and August 2009, a total of 180 pulmonary squamous carcinoma and adenocarcinoma patients from the 1st Affiliated Hospital of Jinzhou Medical University (Jinzhou, China) who were receiving complete pulmonary resection and systematic lymph node dissection were enrolled. During the enrollment period, all patients were pathologically diagnosed for the first time as having NSCLC. Based on the International Association for the Study of Lung Cancer TNM classification system, all of the patients with NSCLC were classified into TNM stages (10). The enrolled patient were required to have integrated clinicopathological information and follow-up data. Strictly based on the National Comprehensive Cancer Network Clinical Practice Guidelines on NSCLC, all of the patients received postoperative treatment (11). Exclusion criteria were as follows: patients receiving preoperative anticancer treatment (including neo-adjuvant chemotherapy, radiotherapy, or biotherapy), patients with previous or simultaneous cancers, patients who died within 1 month after surgery or died from non-cancer diseases.

Until August 30, 2014, all of the patients were followed up. In the retrospective study, patients who were still alive after the last follow-up were censored. Overall survival (OS) means the period between the time of surgery and the last follow-up or death.

### Immunohistochemistry (IHC) and Scoring

Monoclonal mouse anti-HDAC10 antibody (Abcam, No. ab108934) and polyclonal rabbit anti-PD-L1 antibody (Abcam, No. ab213524) for IHC of NSCLC tissues were purchased from Abcam Inc. 180 samples of normal lung tissue were taken from tissue that was 10 cm from the cancer, and were used as para-cancer samples. In line with the viewpoints of two pathologists who were blinded to identity of the NSCLC tissues. Positive staining of HDAC10 and PD-L1 is taken to show the nuclear or cytoplasmic staining of tumor cells. Considering both the staining intensity and the proportion of cells stained, the IHC score was determined by a semi-quantitative method (12). The expression of analyzed makers were assessed using a semi-quantitative method, based on the intensity of color reaction (on a scale of + to +++), and the rate of immunopositive cells (within the ranges of 1–20%, 21–40%, 41–60%, 61–80%, 81–100%) (13). Based on these two variables, a numerical ratio of the markers expression was estimated and used for further analyses. The expression level was further classified into low expression (≤1), moderate expression (1.5-6) and strong positive expression (≥7.5) for both HDAC10 and PD-L1.

### Database for HDAC10 and PD-L1 Expression in Patients With NSCLC

To provide evidence for our findings, we also searched HDAC10 expression in the Oncomine database (14). A total of 226 patients with NSCLC and 20 normal lung tissues were analyzed. Based on the Protein Atlas database (15), the expression of CD274 (PD-L1) in various types of cancer was also investigated. To verify the correlation of HDAC10 and CD274 in patients with NSCLC, an R2 database was used to analyze the relationship of HDAC10 and PD-L1 (16).

### Statistical Analysis

SPSS22.0.0 software was used to perform statistical analysis. The correlation of HDAC10 and PD-L1 expression was analyzed by Spearman's correlation method. OS (Overall Survival) was calculated by the Kaplan-Meier method and analyzed by log-rank test. Based on Cox's proportional hazard model, multivariable analysis of as an independent factor for survival was investigated. *P* < 0.05 was considered statistically different.

## Results

### Expression Level of HDAC10 and Clinicopathological Features of all Patients With NSCLC

**Figure 1:**
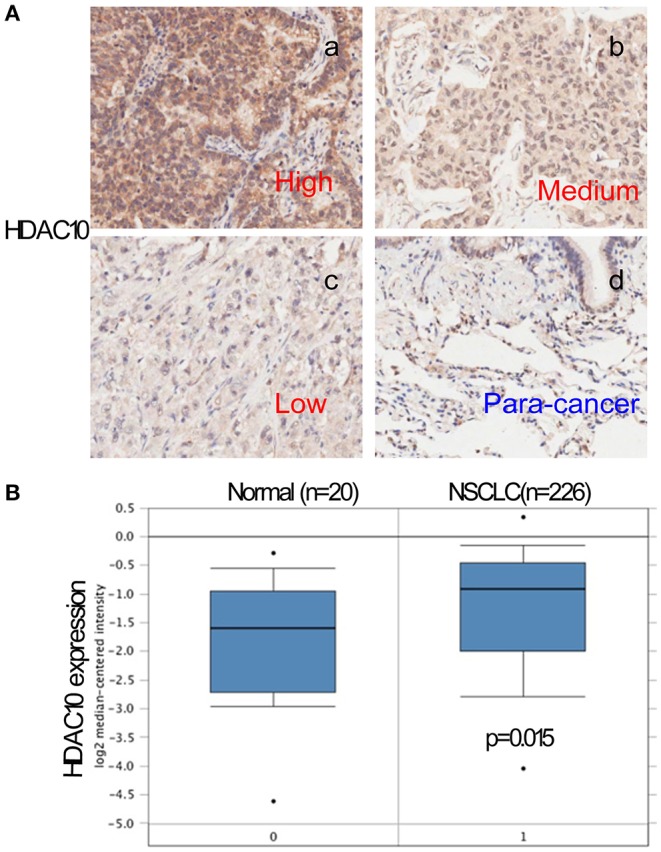
The HDAC10 expression in NSCLC tissues is higher than that in normal lung tissues. **(A)** Tumor tissue and para-cancer lung tissue were stained with HDAC10 antibody. Using both the IHC staining intensity and the proportion of cells stained, the IHC score was determined by a semi-quantitative method. The expression level was further classified into low expression (≤1), moderate expression (1.5-6) and high positive expression (≥7.5) for HDAC10. A representative image of high-, medium- and low-level expression of HDAC10 in 180 patients with NSCLC receiving pulmonectomy (a, b, c), and HDAC10 expression in its corresponding para-cancer tissues (d) were shown. **(B)** Oncomine database for HDAC10 expression in NSCLC tissue (*n* = 226) and normal lung tissue (*n* = 20).

The clinicopathological features of all examined cases are shown in Table 1. Out of 180 NSCLC cases, 64 (35.6%) patients were classified in the high-level of the HDAC10 group. No significant differences were observed between HDAC10 expression and patient characteristics (gender, age, tumor size, and stages). Positive immunostaining of HDAC10 was mainly located in the nucleus and cytoplasm of carcinoma cells (Figure 1A). Noticeably, we observed that the expression score of HDAC10 in NSCLC tissue of 180 patients was significantly higher than that in the corresponding para-cancer tissues (*P* < 0.001). Meanwhile, we searched for HDAC10 expression in patients with NSCLC as compared to normal lung tissue in the Oncomine database. As shown in Figure 1B, the expression level of HDAC10 in NSCLC tissues is 1.55-fold higher than that in normal lung tissues (*p* = 0.020, data were obtained from https://www.oncomine.org/resource/login.html). This result is consistent with our findings.

**Table 1:** The clinicopathological features and analysis of all examined cases.

**Clinicopathological features**	**OS**					
	**N (%)**	**Median, months**	**UVA**	**MVA**		
			***P***	***P***	***HR***	**95%CI**
Gender			0.79	0.34	1.2	0.36–3.8
Male	100 (55.6)	54				
Female	80 (44.4)	51.5				
Age			0.13	0.12	1.7	0.85–3.6
>60	98 (54.4)	52.5				
≤60	82 (45.6)	56.5				
Size			0.9	0.32	1.0	0.52–2.1
>5	55 (30.6)	53				
≤5	125 (69.4)	56				
T			0.19	0.92	1.6	0.78–3.4
T1	36 (20)	66				
T2	102 (56.7)	54				
T3	32 (17.8)	49.5				
T4	10 (6)	14				
N			0.04	0.53	2.0	1–3.8
N0	74 (41.1)	63.5				
N1	68 (37.8)	56				
N2	32 (17.8)	43.5				
N3	6 (3)	24				
M			0.08	0.77	6.1	0.8–46
M0	178 (98.9)	54				
M1	2 (1.1)	14				
TNM			0.00	0.02	2.4	1.5–3.8
I	54 (30)	81				
II	78 (43.3)	64				
III	46 (25.6)	54				
IV	2 (1.1)	16				
PDL1			0.38	0.76	0.81	0.51–1.3
Low	78 (43.3)	62.5				
Moderate	46 (25.6)	53.5				
High	56 (31.1)	44				
HDAC10			0.00	0.00	2.6	1.5–4.5
Low	56 (31.1)	72				
Moderate	60(33.3)	54.5				
High	64 (35.6)	43				

### High-Level HDAC10 Expression Is Positively Associated With PD-L1 Expression in Patients With NSCLC

**Figure 2:**
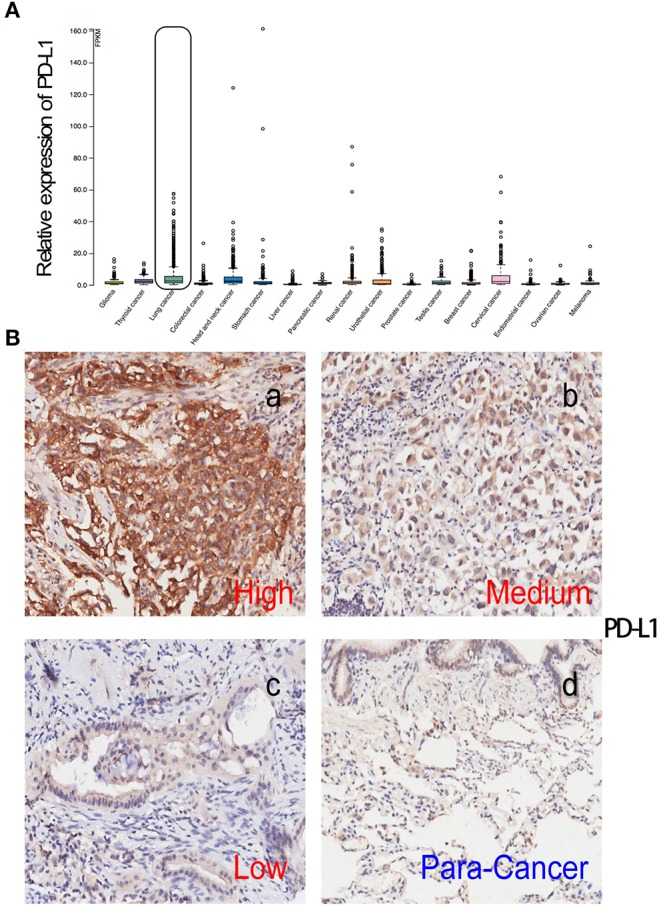
The expression level of PD-L1 in patients with NSCLC. **(A)** The expression level of PD-L1 in all types of cancer, data were from the Protein Atlas website: https://www.proteinatlas.org/ENSG00000120217-CD274/pathology; **(B)** lung tumor tissue and para-cancer lung tissue were stained with PD-L1 antibody. Using both the IHC staining intensity and proportion of cell stained, the IHC score was determined by a semi-quantitative method. The expression level was further classified into low expression (≤1), moderate expression (1.5-6) and strong positive expression (≥7.5) for PD-L1. A reprehensive image of high-, medium- and low-level of PD-L1 expression in tumor tissue from 180 patients with NSCLC receiving pulmonectomy (a, b, c), and PD-L1 expression in its corresponding para-cancer tissue (d).

**Figure 3:**
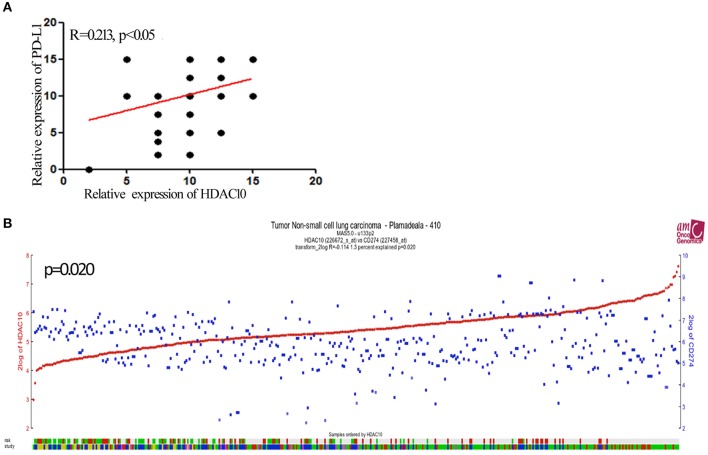
The expression of HDAC10 is positively associated with PD-L1 expression in patients with NSCLC receiving pulmonectomy. **(A)** Correlation between HDAC10 and PD-L1 expression in NSCLC tissues was analyzed. HDAC10 is positively correlated with PD-L1 expression in NSCLC (*r* = 0.213, *p* < 0.05). **(B)** The HDAC10 expression is positively associated with PD-L1 (CD274) in patients with NSCLC (*p* = 0.02). Data were from the R2 website. (https://hgserver1.amc.nl/cgi-bin/r2/main.cgi).

Given that activation of HDACs could regulate PD-L1 expression in myeloma and B-cell lymphomas (17, 18), we further searched for PD-L1 (CD274) in the Protein Atlas website (https://www.proteinatlas.org/ENSG00000120217-CD274/pathology). As seen in Figure 2A, PD-L1 expression in lung cancer is scored at a high-level for all types of cancer. Next, the PD-L1 expression level in NSCLC tissue and normal lung tissue were determined and quantified. The high level of positive staining of PD-L1 was observed in NSCLC tissues, as compared to normal lung tissue (Figure 2B). Univariate and multivariate analysis showed that there is no significant correlation of PD-L1 expression and the prognostic survival after surgery (*P* = 0.76). What is more, the correlation of HDAC10 and PD-L1 expression in NSCLC tissues was analyzed. Patients with high-level expression of HDAC10 often show the overexpression of PD-L1 (Figure 3A). Interestingly, the expression level of HDAC10 is positively correlated with PD-L1 expression in NSCLC tissue (*r* = 0.213, *P* < 0.05). To provide evidence that the positive relationship of HDAC10 and PD-L1 (CD274) in patients with NSCLC, the correlation of these two targets (HDAC10 and CD274) was analyzed (https://hgserver1.amc.nl/cgi-bin/r2/main.cgi). As shown in Figure 3B, the expression level of HDAC10 is positively associated with CD274 expression in patients with NSCLC (p=0.020, n=410). This result further confirmed our findings.

### High-Level HDAC10 Expression Is Associated With a Poor Prognosis in Patients With NSCLC

**Figure 4:**
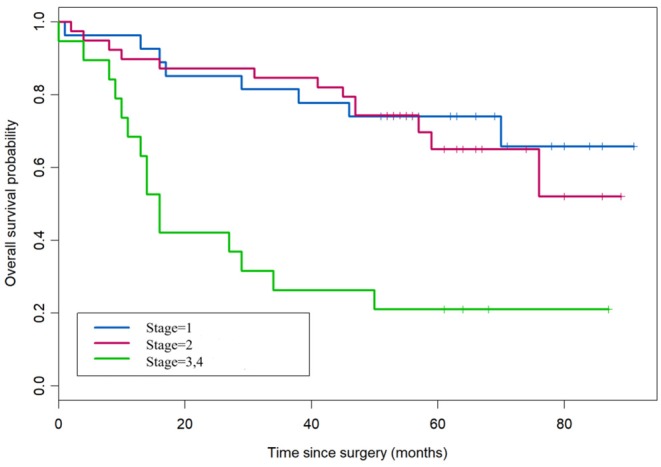
Kaplan-Meier survival curve of patients with NSCLC receiving pulmonectomy. The follow-up time ranges from 22 to 118 months, and the average time is 72 months (median, 48 months). There were 31 deaths and 149 survivors at the last follow-up.

**Figure 5:**
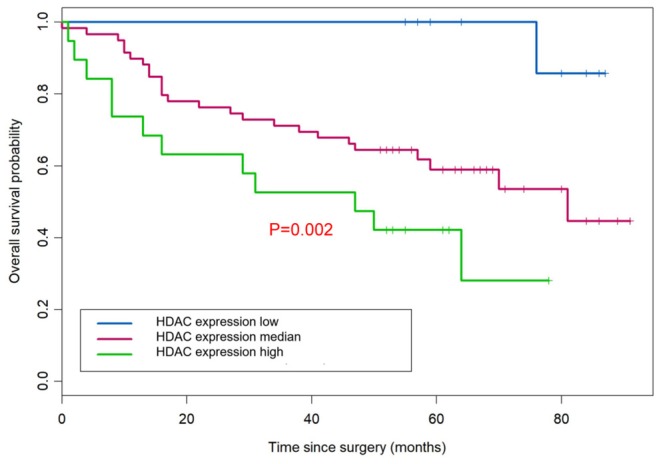
HDAC10 expression is an independent prognostic factor for NSCLC OS. Univariate and multivariate analysis was performed and HDAC10 expression can be used as an independent prognostic factor for OS of NSCLC.

According to the overall survival data from 180 patients with NSCLC, a Kaplan-Meier curve was drawn and is shown in Figure 4. The follow-up time ranges from 22 to 118 months, and the average time is 72 months (median: 48 months). There were 31 deaths and 149 survivors at the last follow-up. We noticed that adenocarcinoma cases are usually at a lower stage than the squamous cell carcinoma cases, reflecting a better outcome of adenocarcinoma than that of squamous cell carcinoma. Furthermore, the result of univariate and multivariate analysis showed that HDAC10 expression is an independent prognostic factor for NSCLC OS. For stage-I and -II NSCLC, patients had shorter OS time than those with stage III (*P* = 0.02, Figure 5). The survival time was significantly different between the groups with high-level and low-level expression of HDAC10 (*P* = 0.002, Figure 5).

In addition, the OS data of 106 patients with NSCLC from the R2 database was analyzed (https://hgserver1.amc.nl/cgi-bin/r2/main.cgi) (19), and the Kaplan-Meier curve suggests that high-level expression of HDAC10 is positively associated with poor prognosis (Figure S4). This result provides proof that HDAC10 could be used as a poor prognosis indicator for patients with NSCLC.

## Discussion

HDAC10, a member of the class II HDAC family, plays a key role in multiple biological processes, including cell proliferation, cell differentiation, cell cycle regulation, homologous recombination, DNA mismatch repair, autophagy, and so on (20–24). Moreover, HDAC10-mediated pathways are closely associated with tumorigenesis and metastasis in various types of cancer. For example, HDAC10 promotes cell proliferation through activation of AKT in lung cancer (25). HDAC10 also regulates the Let-7/HMGA2/Cyclin A2 signaling pathway and further impacts the G_2_/M phase transition in NSCLC (26). In human colorectal cancer, HDAC10 expression is associated with DNA mismatch repair genes. HDAC10 mRNA levels correlate with the platinum sensitivity of ovarian carcinoma cells (27). Suppression of HDAC10 could initiate HSP70-mediated autophagy and activate CMA (chaperone-mediated autophagy) and induce degradation of a CMA substrate in HeLa cells (24). Thus, HDAC10 may be a potential target for the diagnosis and treatment of many cancers.

Recently, researchers have found that class II HDACs (including HDAC10) are associated with poor prognosis independently of any clinicopathologic feature (28). Among the class II HDAC family, *HDAC10* is significantly correlated with poor prognosis in renal cancer, melanoma and gastric cancer patients (6, 29, 30). However, the expression of HDAC10 and its association with clinicopathological features in NSCLC is not clear. Herein, we analyzed 180 NSCLC patients receiving complete pulmonary resection and systematic lymph node dissection from April 2004 to August 2009. We observed that HDAC10 expression in lung cancer tissue is significantly higher than that in corresponding para-cancer tissue. Moreover, the Oncomine database demonstrated that HDAC10 expression in NSCLC tissues is 1.55-fold higher than in normal lung tissues (Figure 1B) (15). This result confirmed that HDAC10 expression in NSCLC tissues is higher than that in normal lung tissues. Our findings suggest that HDAC10 could be a promising biomarker that might provide novel strategies for clinicians to improve efficacy for treatment of NSCLC. Furthermore, based on the overall survival data from 180 patients with NSCLC, a Kaplan-Meier curve showed that HDAC10 expression is an independent prognostic factor for NSCLC OS. Noticeably NSCLC patients in stage-I and -II had a shorter OS time than those with stage-III (Figure 5). The survival time between groups with high-level and low-level expression of HDAC10 had significant differences. Meanwhile, the OS data of 106 patients with NSCLC from the R2 database suggests that high-level expression of HDAC10 is positively associated with poor prognosis (Figure S4). Our result provides proof that HDAC10 could be serve as a poor prognosis indicator for patients with NSCLC.

Tumor-associated PD-L1 (CD274) can block tumor-specific T cell-mediated immunity by inducing apoptosis of T cells, suppressing the secretion of cytokines and disturbing the function of activated T cells (31, 32). PD-L1 expression by tumors and its interaction with PD-1-expressing T cells in the tumor microenvironment can lead to immunotherapy tolerance. Targeting this co-inhibitory axis has proven clinically effective in the treatment of NSCLC (33). Meanwhile, recent findings suggested that activation of HDACs could induce PD-L1 expression in various types of cancer, especially in myeloma and B-cell lymphomas (7, 8, 34). Though the relationship of HDAC10 and PD-L1 expression with poor prognosis in patients with NSCLC is unclear, it is possible that HDAC10 is related to PD-L1 expression in NSCLC. Herein, we found a stronger positive staining of PD-L1 in NSCLC as compared to normal lung tissue. Interestingly, we noticed that HDAC10 is positively correlated with PD-L1 expression in patients with NSCLC. Based on the R2 database, the correlation of these two targets (HDAC10 and CD274) was evaluated. It provides evidence that HDAC10 is positively associated with CD274 expression in NSCLC (p=0.020). Targeting PD-L1 with monoclonal antibodies has significantly impacted the treatment landscape for NSCLC during the last 5 years. Due to the lack of definite biomarkers to select as optimal responders, only about 20% of patients with advanced NSCLC respond to PD-L1-based immunotherapy. Herein, we found that patients with high-level expression of HDAC10 often show PD-L1 overexpression. HDAC10 can be used as a potential biomarker for PD-L1 treatment. In addition, combination approaches to PD-L1-based immunotherapy are currently designed to re-energize the immune system with a synergetic mechanism and could achieve durable antitumor effects in NSCLC. Our findings also suggest the potential combination of PD-L1 inhibitors in NSCLC alongside targeted inhibition of HDAC10.

Given that the HDAC inhibitor (HDACi) pembrolizumab is currently in clinical trials for use in lung cancer patients (35), our findings will be helpful to establish clinical study protocols. What is more, our results suggested that HDAC is might act on the immune networks of lung cancer cells, including changes in the profiles of co-stimulatory molecules, co-stimulatory antigens and cytokines of cancer cells. Meanwhile, our results also indicated that there is an intimate connection between targeting epigenetics and PD-L1-based immunotherapy. Therefore, the expression level of HDAC10 in NSCLC might predict the therapeutic response to HDAC inhibitors. Similar to other targeted therapeutics, treatment response might be especially prominent in patients with overexpressed HDAC10 (28, 36). In addition, there were two limitations in this study. Firstly, we investigated the expression of HDAC10 and PD-L1 mostly in patients with TNM stage I to IIIa who had the opportunity to receive surgical therapy. For TNM stage-IV NSCLC patients, the data is unavailable. Secondly, our results suggest the connection of HDAC10 and PD-L1, while the precise mechanisms of PD-L1 expression in NSCLC will be addressed in the future.

## Conclusions

Taken together, our findings suggest clinical relevance for HDAC10 and PD-L1 (CD274) expression and provide a rationale strategy for the combination of PD-L1 blockade and HDAC10 inhibition.

## Data Availability Statement

The datasets generated for this study are available on request to the corresponding author.

## Ethics Statement

This study was carried out in accordance with the recommendations of 1st Affiliated Hospital of Jinzhou Medical University Medical Ethics Committee. The protocol was approved by the 1st Affiliated Hospital of Jinzhou Medical University Medical Ethics Committee. All subjects gave written informed consent in accordance with the Declaration of Helsinki (Figures S1–S3).

## Author Contributions

XL and YZ performed the experiments. YuW, RZ, TJ, LQ, QJ, JZ, JS, ZW, LW, TL, XM, YiW, and NW participated in the experiments and drafted the manuscript.

## Conflict of Interest

The authors declare that the research was conducted in the absence of any commercial or financial relationships that could be construed as a potential conflict of interest.

## Supplementary Material

Figure S1 Proof of medical ethics.

Figure S2 Approve of ethics committee.

Figure S3 Biomedicine research informed consent form.

Figure S4 Kaplan-Meier survival curve of NSCLC patients with high- and low-HDAC10 expression. The data was obtained from the R2 dataset. (https://hgserver1.amc.nl/cgi-bin/r2/main.cgi).
