# Supplementary material for: HDAC10 Is Positively Associated With PD-L1 Expression and Poor Prognosis in Patients With NSCLC
Source: Front Oncol. 2020 Apr 21;10:485. doi: 10.3389/fonc.2020.00485 (PMC7186423; doi:10.3389/fonc.2020.00485)
Supplement: Figure S1 — Proof of medical ethics. [file Presentation_1.PPTX]

## Slide 1
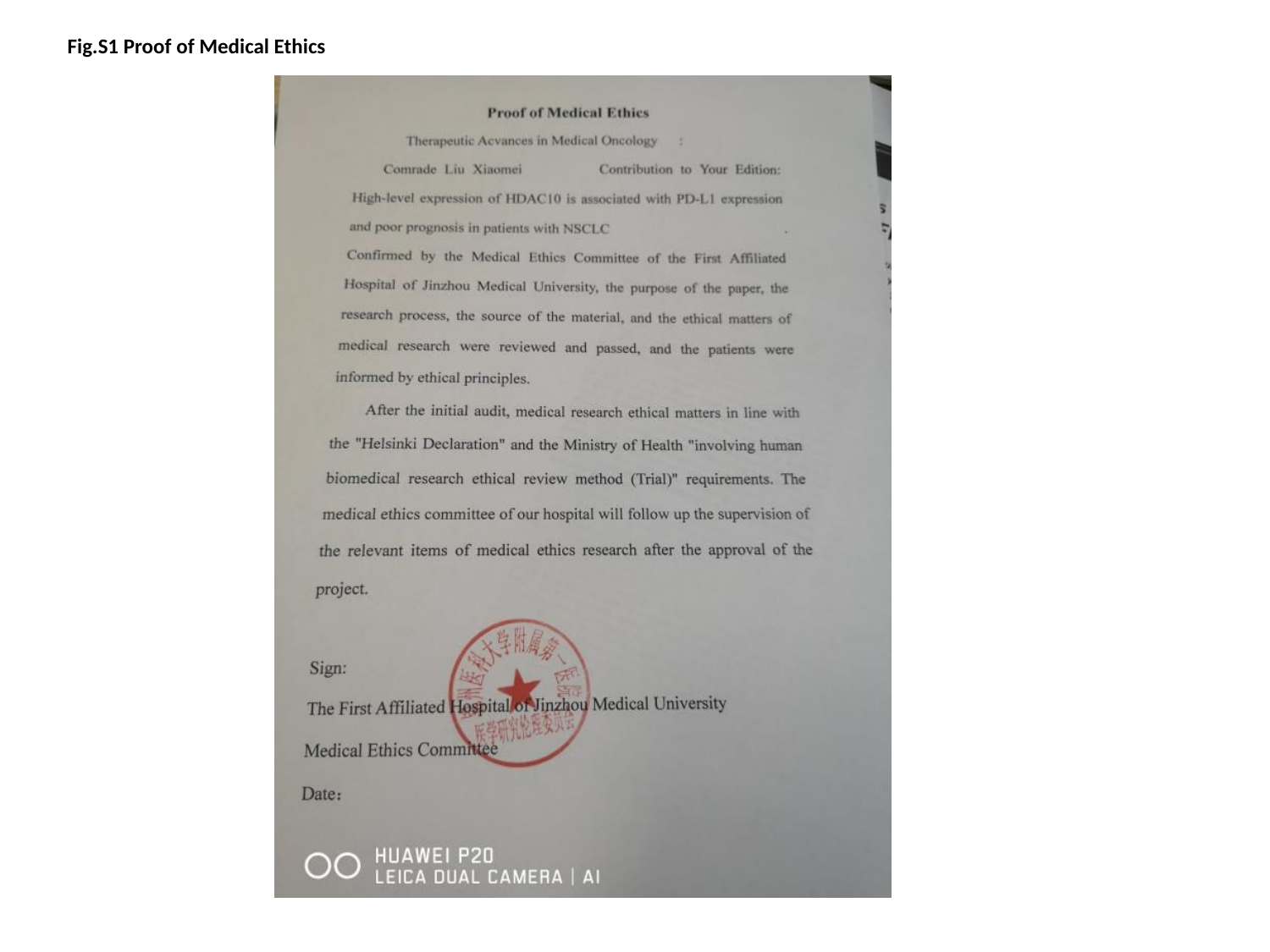

Fig.S1 Proof of Medical Ethics

## Slide 2
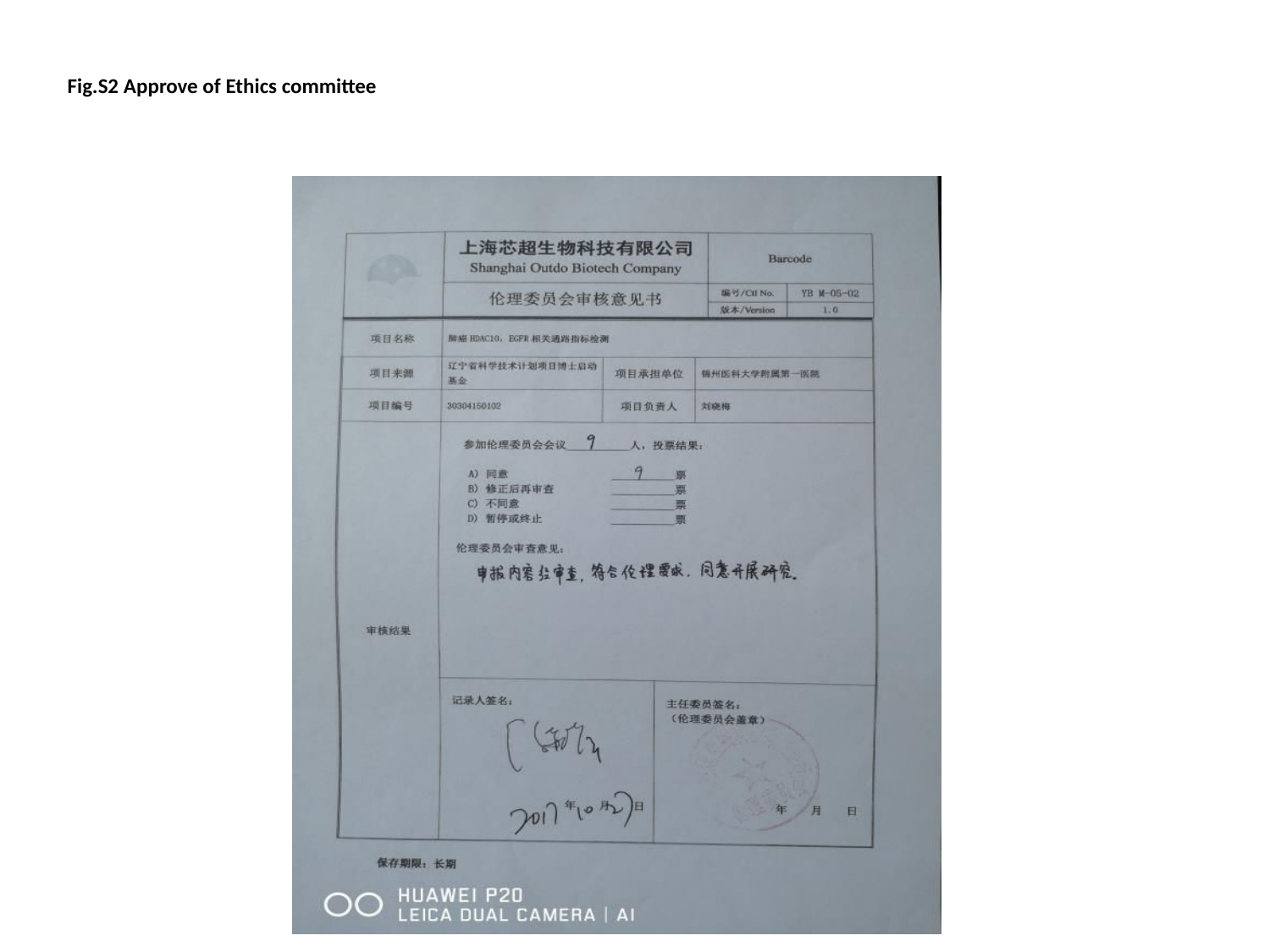

Fig.S2 Approve of Ethics committee

## Slide 3
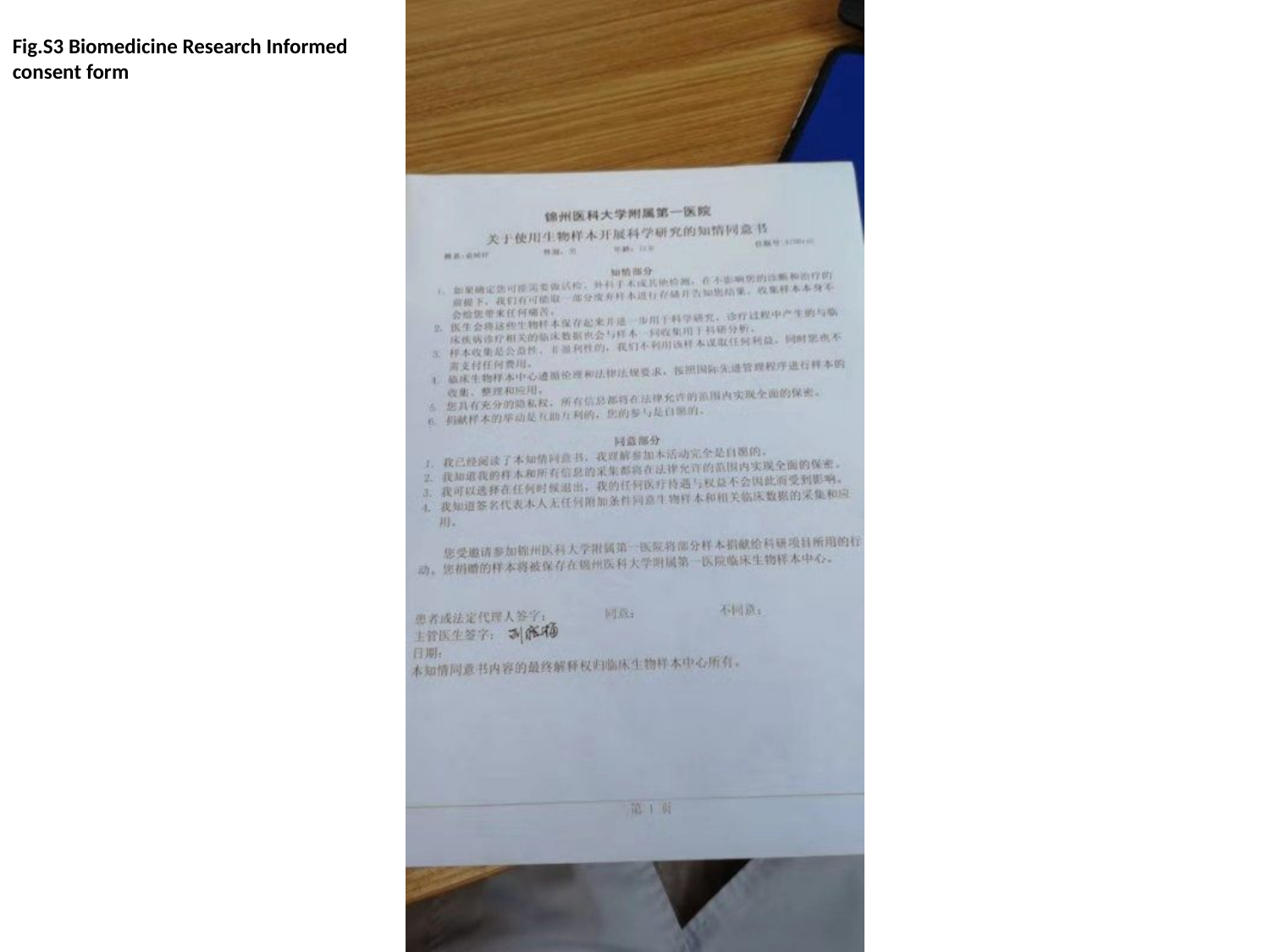

Fig.S3 Biomedicine Research Informed consent form

## Slide 4
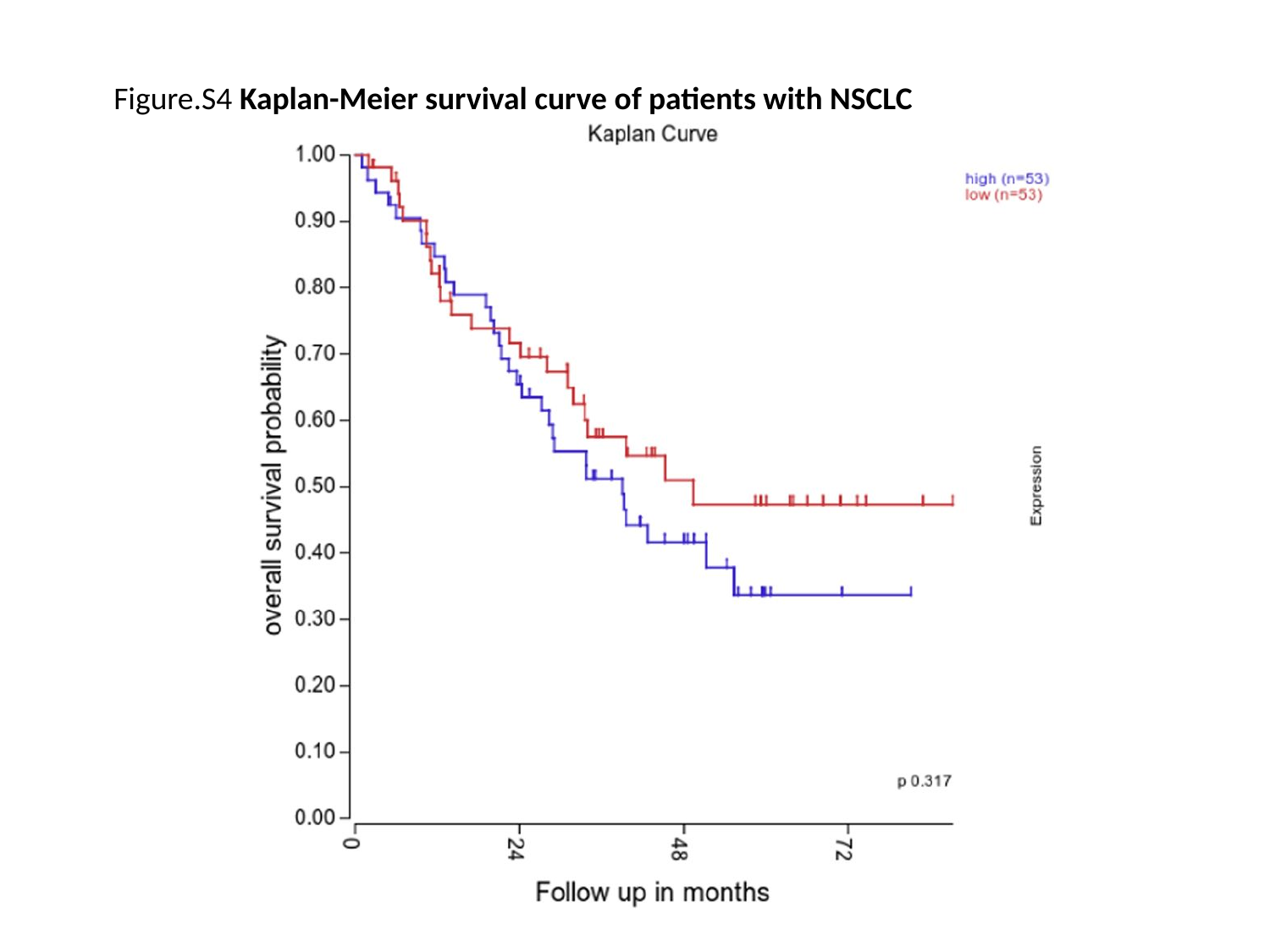

Figure.S4 Kaplan-Meier survival curve of patients with NSCLC
